# Sudden death of an infant with cardiac, nervous system and genetic involvement – a case report

**DOI:** 10.1186/1746-1596-8-159

**Published:** 2013-09-20

**Authors:** Donatella Mecchia, Valentina Casale, Roberta Oneda, Luigi Matturri, Anna Maria Lavezzi

**Affiliations:** 1 “Lino Rossi” Research Center for the study and prevention of unexpected perinatal death and SIDS - Department of Biomedical, Surgical and Dental Sciences, University of Milan, Via della Commenda 19, Milan 20122, Italy

**Keywords:** SIDS, Hypertrophic cardiomyopathy, Mahaim fiber, Raphe system, Serotonin transporter gene polymorphism

## Abstract

**Abstract:**

We present a case of sudden death of a 1-month-old male infant with heart, brainstem and genetic polymorphism involvement. Previously considered quite healthy, the child died suddenly and unexpectedly during sleep. The autopsy protocol included an in-depth anatomopathological examination of both the autonomic nervous system and the cardiac conduction system, and molecular analysis of the serotonin transporter gene promoter region, in which a specific genetic condition seems to be associated with sudden infant death. Histological examination revealed the presence of congenital cardiac alterations (hypertrophic cardiomyopathy and an accessory Mahaim fiber in the cardiac conduction system), severe hypodevelopment of all the raphe nuclei and a heterozygous genotype L/S related to the serotonin transporter gene. The sudden death of this infant was the unavoidable outcome of a complex series of congenital anomalies, each predisposing to SIDS.

**Virtual slides:**

The virtual slide(s) for this article can be found here: http://www.diagnosticpathology.diagnomx.eu/vs/3480540091031788

## Background

Sudden infant death syndrome (SIDS) is a terrible event defined as a sudden, unexpected death that remains unexplained after careful study, and represents a multifactorial problem [1,2]. SIDS strikes one infant every 700–1,000 live births, being the most frequent cause of death within the first year of life [3]. In 2007 the International Stillbirth Alliance and more recently the USA Academy of Pediatrics [4,5] stressed the need to submit all victims of such deaths to neuropathologic studies. In fact, the possibility of preventing perinatal unexpected death and SIDS relies mainly on a better knowledge of the underlying alterations of the central autonomic nervous system (ANS), and of the cardiac conduction system (CCS), which functions under the control of the nervous system [6].

Our in-depth histopathological and immunohistochemical examinations performed on a wide group of sudden death victims [7] have revealed the presence of many neuromorphological and/or functional alterations, such as hypoplasia of different structures of the brainstem, spinal cord and cerebellum (arcuate nucleus, reticular formation with the pre-Bötzinger nucleus, hypoglossus nucleus, Kölliker-Fuse nucleus, parafacial complex, intermediolateral nucleus, cerebellar cortex), lack of tyrosine-hydroxylase in neurons of the locus coeruleus and abnormal synthesis of somatostatin in the hypoglossus nucleus. As to the cardiac conduction system, developmental alterations such as accessory pathways (mainly Mahaim fibers), atrioventricular node duplicity, meta-hyperplasia of the central fibrous body, can be anatomic substrata for reentrant pathways, dissociation of the impulse conduction, and paroxysmal tachycardias [6,8]. All these alterations of the autonomic nervous system and of the cardiac conduction system, that are frequently associated, arise from a common genetic background.

Genes investigated as possible genetic predisposing factors for SIDS include the serotonin transporter gene promoter region (*5-HTT*) [9]. In particular, an association between the more effective promoter long allele (L) polymorphism of the *5-HTT* gene and the risk of SIDS has been demonstrated.

Below, we report a case of sudden death of a 1-month-old infant, that is noteworthy because of the combination of multiple developmental alterations. Anatomopathological and genetic examination revealed, in fact, the presence of hypertrophic cardiomyopathy associated to accessory fibers in the cardiac conduction system, severe hypoplasia of all the brainstem raphe nuclei and a heterozygous genotype for *5-HTT* (L/S).

## Case presentation

The infant was a 1-month-male, born at 41 weeks of gestation and with a birth weight of 3500 g. He was in apparent good health until the day he died, suddenly and unexpectedly in his crib. His parents declared that so far as they knew, he had suffered from no illness or complaint of any nature. There was no significant family history relevant to the case, except for heterozygous factor V Leiden in the mother. The mother had had a previous pregnancy in 2010 and the baby had always been healthy. The mother was asked about drugs or alcohol abuse and smoking habit before, during and after pregnancy, but she denied using any of these substances known to be risk factors.

## Methods

A complete autopsy was performed according to the protocol followed by our Research Center in cases of sudden death [10], also available on the web site http://users.unimi.it/centrolinorossi/en/guidelines.html. The guidelines include, in particular, an in-depth anatomopathological examination of the autonomic nervous system and of the cardiac conduction system. In addition, a fresh specimen of cerebral cortex is required for genetic analysis of the *5-HTT* polymorphism.

Multiple samples of all organs were fixed in 10% formalin buffer, processed, and embedded in paraffin. Five-micrometer sections were stained with hematoxylin and eosin.

### Cardiac conduction system examination

The heart was weighed and measured, and the values were compared with the normal values for infants of that length and age. Then it was fully examined for pathological changes in the atria, septa, ventricles, pericardium, endocardium, and coronary arteries. Samples of the myocardium were stained with hematoxylin and eosin and trichromic Heidenhain (azan). Histological observations were focused on the cardiac conduction system, which was removed in two blocks, according to our guidelines: the first included the sino-atrial node (SAN) and the “crista terminalis”, the second contained the atrioventricular node (AVN), His bundle, and bundle branches. The samples were stained alternately with hematoxylin/eosin and trichromic Heidenhain (azan).

### Central autonomic nervous system examination

Specimens from the brainstem were collected, processed and embedded in paraffin. Transverse serial sections from all the samples were made at intervals of 60 μm. For each level, twelve 5 μm sections were obtained, two sections were routinely stained for histological examination using hematoxylin-eosin and Klüver-Barrera stains. Additional sections were saved and stained as deemed necessary for further investigations. The main nuclei were analyzed: the parafacial, the retrotrapezoid, the locus coeruleus, the Kölliker-Fuse nuclei and the rostral group of the raphe nuclei (median, magnus, dorsal, caudal linear nuclei) in the pons and mesencephalon; the hypoglossus, the dorsal motor vagal, the tractus solitarius, the ambiguus, the pre-Bötzinger, the inferior olivary, the arcuate nuclei and the caudal group of the raphe nuclei (obscurus and pallidus nuclei) in the medulla oblongata.

### Genetic analysis of the polymorphic region of the serotonin transporter gene

#### DNA isolation

DNA was isolated by the specific fresh specimen conserved in RNA-later reagent (AMBION, Inc; Austin, TX), using Invisorb® Spin Tissue Mini Kit (INVITEK, GmbH, Germany) and following the manufacturer instructions.

#### Genotype and allele analysis

The 5-HTT polymorphism was genotyped using specific primers according to Heils et al. [11] (forward: 5-GGCGTTGCCGCTCTGAATGC-3; reverse: 5-GAGGGACTGAGCTGGACAACCAC-3).

PCR was carried out in a final volume of 50 μl consisting of approximately 50 ng genomic DNA. Amplification was performed using: 20 μl of genomic DNA, 1× PCR Gold buffer (Applied Biosystems, Foster City, USA), 2 mM MgCl2, 0.4 mM dNTPs, 50 pmol each of the required primers and 2U AmpliTaq Gold (Applied Biosystems). Temperature cycling was performed using an Applied Biosystems 2720 Thermal Cycler with the following new protocol which was performed in our laboratory: 10 min at 95°C, followed by 40 cycles of 95°C for 1 min and 61°C for 10 min, with a final extension for 7 min at 72°C. PCR amplification products were electrophoresed through a 1,5% agarose gel and visualized by UV-light in presence of ethidium bromide.

## Results

**Figure 1:**
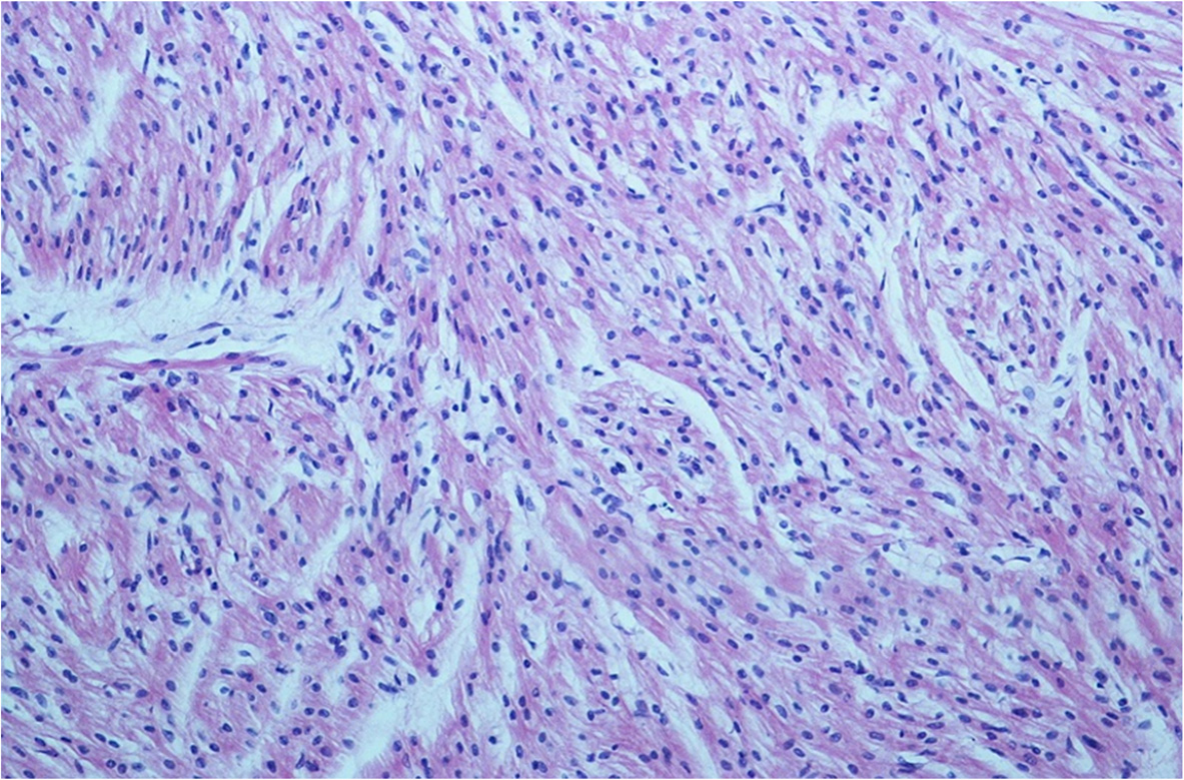
**Hypertrophy of myocytes and cellular disorganization (disarray) of the left ventricle.** Hematoxylin and Eosin stain; magnification 40 ×.

**Figure 2:**
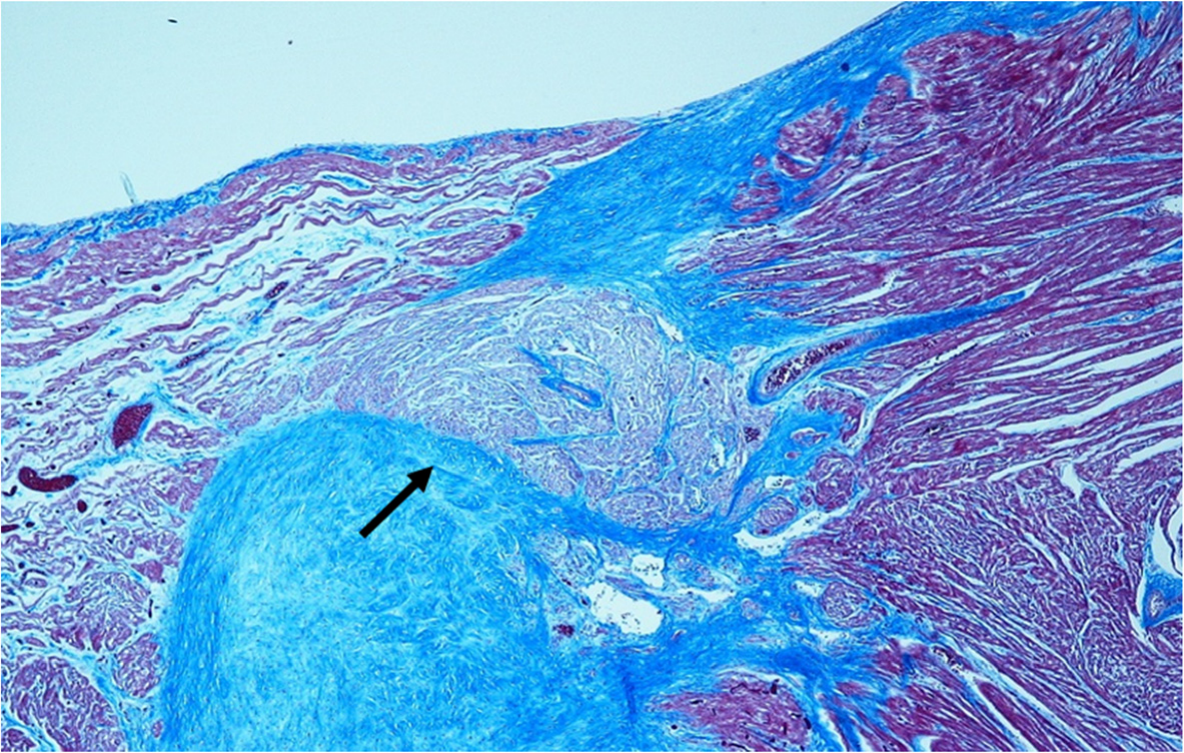
**The accessory nodo-ventricular fiber of Mahaim (narrow) and cartilaginous metaplasia of the cardiac fibrous body (a).** Azan stain, magnification 10 ×.

At autopsy, the infant was described as in good health, with a body length (54 cm) and weight (4500 gr) at the 50th percentile. There were no signs of violence. The lungs were congested, with some alveolar and interstitial bacterial colonies, edema and many intra-alveolar lipid vacuoles, suggestive of a lipid pneumonia due to aspiration. The heart, that arrived without the tip, weighed 27 gr; the cardiac diameters were as follows: transverse 4.1 cm and anteroposterior 2.9 cm. We could not measure the longitudinal diameter due to the absence of the tip. The myocardium was brownish and homogeneous in appearance, with clots in the left ventricle. The foramen of Botallo was open by about 3 mm. The coronaries appeared normal. Histological examination of the common myocardium showed congestion, myocytes hypertrophy and cellular disorganization (disarray) of the left ventricle and of the interventricular septum (Figure 1). Histological examination of the cardiac conduction system showed an accessory fiber of Mahaim (nodo-ventricular) and cartilaginous metaplasia of the cardiac fibrous body (Figure 2).

**Figure 3:**
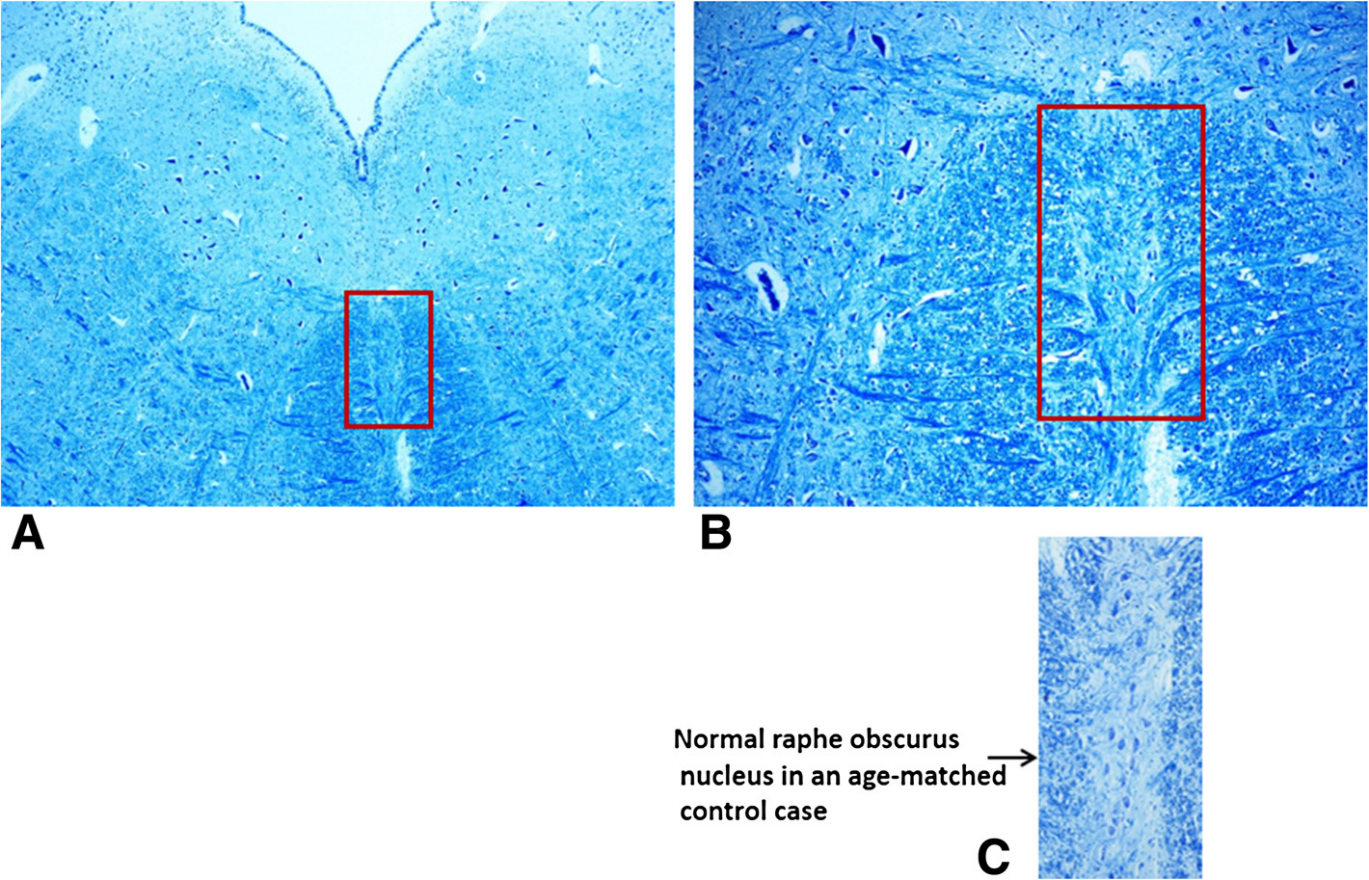
**Hypoplasia of the raphe obscurus nucleus.** The surrounded area in **A)** is represented at higher magnification in **B)**. In **C)** a normal raphe obscurus nucleus in an age-matched control case. Klüver-Barrera stain; magnification: **A)** 4 ×; **B) **20 x; **C)** 10 ×.

The histological examination of the brainstem highlighted severe hypoplasia of all the serotonergic nuclei of both the rostral and the caudal raphe system (dorsal, median and caudal linear nuclei in the rostral group; magnus, obscurus and pallidus nuclei in the caudal group) (Figure 3).

Molecular analysis of the polymorphic region of the serotonin transporter gene demonstrated that the infant was heterozygous for the *5-HTT* gene with the presence of both the short (S) and the long (L) allele in the genotype.

The final diagnosis was: left ventricle and interventricular septum hypertrophic cardiomyopathy; a nodo-ventricular accessory Mahaim fiber in the cardiac conduction system; severe hypoplasia of all the nuclei of the serotonergic raphe system in the brainstem; a polymorphic L/S genotype of the serotonin transporter gene promoter region. Table 1 provides all clinical and pathologic findings related to this case.

**Table 1:** Clinical and pathologic features of the case report

Age	1 month
Sex	Male
Pregnancy	Regular
Week of gestation at birth	41
Weight at birth	3500 gr
Relevant prior history	None
Feeding	Breast-feeding
Usual position in sleep	Supine
Maternal smoking	No
Maternal drug/alcohol abuse	No
Autoptic examination	
Heart	Hypertrophic cardiomyopathy
	Accessory fiber of Mahaim in the cardiac conduction system
Brain	Rapfe nuclei hypoplasia
Genetic analysis of the 5-*HTT* gene	Polymorphic L/S genotype

## Discussion

Different pathologies can be the cause of a sudden unexpected death, such as congenital malformations, cardiomyopathies and vascular hyaline [12-14]. Undiagnosed congenital heart diseases are in particular very frequent in paediatric autopsies [15,16]. In the case of sudden neonatal death here presented a congenital cardiomyopathy was associated with brainstem developmental alterations and polymorphism of the serotonin transporter gene.

These findings primarily confirm the link we have previously highlighted in a large group of perinatal sudden deaths (28 SIDS and 12 SIUDS victims) between neuropathological raphe defects and serotonin transporter promoter region polymorphisms [17]. In particular, we have suggested the presence of the L allele as a predisposing factor for morphological developmental defects of the brainstem raphe nuclei leading to sudden death.

It is well known that the neurons of the raphe nuclei, producing the neurotransmitter serotonin, besides playing a trophic role during neuronal development in the fetal brain [18], are involved in the breathing mechanism after birth, modulating the ventilatory responses to hematic oscillations of pO_2_, pCO_2_ and pH in order to maintain these within physiological levels [19]. We may speculate that the severe hypoplasia of all the raphe nuclei that we observed in this case, plausibly a consequence of the genotypic presence of the *5-HTT* L allele, may have prevented eupneic breathing, so leading to death.

Furthermore, in our previous study [17] we indicated that hypoplasia of the raphe nuclei and the L/L or S/L genotypes are significantly associated to maternal smoking in pregnancy. In support of this concept, experimental studies in rats have shown that prenatal nicotine absorption can directly act on the expression of serotonin transporter genes [20]. In this case, however, the mother admitted no history of cigarette smoking. Nevertheless, it should be considered that a retrospective assessment of the maternal smoking habit, mainly if performed after the fatal event, is sometimes unreliable [21]. Smoker mothers are generally reluctant to honestly report tobacco use, because of feelings of guilt.

The neuropathological and genetic features observed in this case were exacerbated by the additional presence of accessory fibers in the cardiac conduction system and of hypertrophy of the heart myocytes. The Mahaim fiber is an accessory atrio-ventricular communication quite frequent in perinatal unexplained death, that, under particular conditions and/or neurovegetative stimuli, is liable to provoke electrical dyshomogeneity, instability and desynchronization, raising the risk of malignant functional arrhythmias.

Hypertrophic cardiomyopathy (HCM) is the most prevalent cause of sudden cardiac death in younger people, frequently determined by mutations in genes coding for proteins of the sarcomere [22]. Neonatal clear HCM is, instead, a very rare observation, being a progressive heart disease that, though present from birth, develops over time. In a retrospective study performed on DNA extracted from paraffin blocks tissues of a wide group of SIDS victims, Brion et al. [23] observed four different polymorphic genes associated to HCM (namely, *MYBPC3; TNNT2; MYH6; TNN13*) in 10% of cases, without pathological manifestations. Thus, the diagnosis of a clearly evident HCM in the infant here presented reinforces the importance of this report. Research aimed at evaluating the expression of specific HCM genes is now in progress in our laboratory in order to verify whether multiple genetic variants may be at the basis of this complex series of congenital anatomopathologic findings.

In conclusion, we cannot define this report as a true SIDS case, since SIDS is defined as a sudden infant death that remains undetermined after all investigative avenues have been exhausted, but given the combination of multiple pathological events underlying the sudden death, as a border-line SIDS case it does, however, contribute to provide further insights into this tragic phenomenon.

## Consent

Parents of the infant provided written informed consent to both autopsy and genetic study, under protocols approved by the Milan University L. Rossi Research Center institutional review board. Written informed consent was obtained from the parents also for publication of this Case Report.

## Competing interests

The authors declare that they have no competing interests.

## Authors’ contributions

LM conceived the study. AML was responsible of the neuropathological examination and DM of the heart examination. VC and RO performed the genetic analysis. All authors participated in the writing of the manuscript and approved the final version.
